# Genetic biomarkers related to hemarthrosis, inflammation, and cartilage structure in pediatric patients with hemophilic arthropathy

**DOI:** 10.1002/mgg3.979

**Published:** 2019-09-30

**Authors:** José de Jesús López‐Jiménez, Ricardo Ortega‐Cervantes, Hilda Luna‐Záizar, Ana‐Lilia Fletes‐Rayas, Claudia‐Patricia Beltrán‐Miranda, Rogelio Troyo‐Sanromán, Janet Soto‐Padilla, Alberto Tlacuilo‐Parra, Ana‐Rebeca Jaloma‐Cruz

**Affiliations:** ^1^ División de Medicina Molecular Centro de Investigación Biomédica de Occidente IMSS DFMI, Centro Universitario de Ciencias de la Salud (CUCS), Universidad de Guadalajara Guadalajara Jalisco México; ^2^ Servicio de Ortopedia Unidad Médica de Alta Especialidad Hospital de Pediatría Centro Médico Nacional de Occidente Guadalajara Jalisco México; ^3^ Departamento de Química Centro Universitario de Ciencias Exactas e Ingenierías Universidad de Guadalajara Guadalajara Jalisco México; ^4^ Departamento de Enfermería Clínica Integral Aplicada Centro Universitario de Ciencias de la Salud (CUCS) Universidad de Guadalajara Guadalajara Jalisco México; ^5^ Centro Universitario del Sur Universidad de Guadalajara Ciudad Guzmán Jalisco México; ^6^ Departamento de Fisiología Centro Universitario de Ciencias de la Salud (CUCS), Universidad de Guadalajara Guadalajara Jalisco México; ^7^ Servicio de Hematología Unidad Médica de Alta Especialidad Hospital de Pediatría Centro Médico Nacional de Occidente Guadalajara Jalisco México; ^8^ División de Investigación Médica Unidad Médica de Alta Especialidad Hospital de Pediatría Centro Médico Nacional de Occidente Guadalajara Jalisco México; ^9^ División de Genética Centro de Investigación Biomédica de Occidente IMSS Guadalajara Jalisco México

**Keywords:** diagnostic‐imaging, genetics, hemarthrosis, hemophilia, joint‐diseases, population

## Abstract

**Background:**

The pathophysiology of hemophilic arthropathy is complex and not completely understood. In this study, we aimed to identify biomarkers that can affect the hemophilic arthropathy severity.

**Methods:**

Fifty patients were analyzed for biomarker frequencies; in 37 patients, articular symptoms were evaluated based on the physical joint examination score, and in 18, it was based on magnetic resonance imaging. Eight polymorphisms, namely *FV 1691G>A*, *FII 20210G>A, MTHFR 677C>T* and *1298A>C*, *TNFα‐308G>A* and ‐*238G>A*, *ACAN* VNTR, and IL1RN*2‐VNTR were identified.

**Results:**

Patients with the *MTHFR 677TT* genotype showed a higher number of affected joints (1.83 ± 0.9 vs. 0.55 ± 0.7 for *CC*; *p* = .023), whereas those with the *MTHFR 1298AC* genotype exhibited higher effusion according to two radiologists (0.90 ± 0.31/1.20 ± 0.63 vs. 0.38 ± 0.52/0.50 ± 0.53 for *AA* genotype; *p* = .043/0.036, respectively). In addition, patients with the *TNFα‐308GA* genotype had more subchondral cysts (0.75 ± 0.95 vs. 0.07 ± 0.26 for *GG* genotype; *p* = .041).

**Conclusions:**

The distribution of risk genotypes for *MTHFR* and *TNFα‐308GA* suggests their association with clinical parameters of hemophilic arthropathy. Cohort studies are essential to verify these associations.

## 1 INTRODUCTION

Hemophilia A (HA) and B (HB) are an X‐linked recessive disease caused by mutations in *F8* or *F9* resulting in deficiency of coagulation factors (F) VIII or IX, respectively. A major symptom is recurrent hemarthrosis and subsequent progressive arthropathy (Roberts & Hoffman, 2001) aggravated by inflammation (Forsyth et al., 2012). Hemophilic arthropathy is histologically manifested as synovial hyperplasia with increased vascularity, discoloration due to hemosiderin deposits, villus formation, and irreversible cartilage erosion with chondrocyte apoptosis (Valentino et al., 2012). Surprisingly, articular damage varies in severity and in some patients can occur despite prophylaxis, whereas in others, it was not observed even after multiple episodes of hemarthrosis (Valentino et al., 2012). Magnetic resonance imaging (MRI), is the gold standard method for detailed evaluation of hemophilic arthropathy (Lundin et al., 2012). However, MRI is expensive, whereas articular physical examination of joint function is affordable (Hacker, Funk, & Manco‐Johnson, 2007). In this study, we assessed the severity of hemophilic arthropathy by MRI and physical examination, and explored its association with genetic biomarkers linked to thrombophilia, including the following single nucleotide substitutions, four predicting missense variants and one in the 3′‐UTR region (F2): “*FV 1691G>A*”, NM_000130.4:c.1601G>A, NP_000121.2:p.Arg534Gln (dbSNP: rs6025) on *F5* coding sequence (CDS) (Chr1); “*FII 20210G>A*”, NM_000506.4:c.*97G>A, NG_008953.1:g.25313G>A (dbSNP: rs1799963) on *F2* (Chr11); “*MTHFR 677C>T*”, NM_005957.4(MTHFR):c.665C>T, NG_013351.1:g.14783C>T (dbSNP: rs1801133); “*MTHFR 1298A>C*”, NM_005957.4(MTHFR):c.1286A>C, NG_013351.1:g.16685A>C (dbSNP: rs1801131), the last two being in *MTHFR* CDS (Chr1). We also considered the genetic biomarkers linked to inflammation, two single nucleotide substitutions and one VNTR (*IL1RN*): “*TNFα‐308G>A*”, TNF (NG_007462.1):g.4752G>A; TNF (NM_000594.3): c.‐418G>A (dbSNP: rs1800629); “*TNFα‐238G>A*”, TNF (NG_007462.1):g.4682G>A; TNF (NM_000594.3): c.‐488G>A (dbSNP: rs361525), both on the *TNF* promoter (Chr6); “IL1RN*2 VNTR”, NM_173841.2:c. (dbSNP: rs2234663) consisted of 4 intronic repeats of an 86‐bp (Chr2) core sequence (four alleles ranging from 2 to 5 repeats), in which the shortest allele (i.e., IL1RN*2) was associated with high gene expression and increased IL‐1RA plasma levels. We explored its association with genetic biomarkers linked to cartilage compression resistance. The “A*CAN* VNTR”, NG_012794.1 (variant of cartilage‐specific proteoglycan aggrecan) contained 13 alleles ranging from 17 to 31 repeats of a 57‐bp core sequence in the CDS (Chr15) of *ACAN* exon 12 (the reference sequence contains 26 repeats), and shorter alleles associated with a reduced resistance to compression in the cartilage, also possibly associated with higher risks of joint damage.

## 2 MATERIALS AND METHODS

### 2.1 Study population

Fifty patients were analyzed for biomarker frequencies. The clinical data were collected from 2007 to 2010 for hemophilic patients from western Mexico who underwent timely on‐demand treatment within 1–2 hr after the onset of major hemorrhage (FVIII 20–30 IU/kg or FIX 40–50 IU/kg every 12 hr) (Esparza‐Flores, 2005); patients with moderate and mild hemophilia eventually received 0.3 μg/kg desmopressin intranasally. Patients with present or past positivity for coagulation factor inhibitors were excluded. One patient developed inhibitors during the analysis and was, therefore, excluded. Overall, 43 children with HA and six with HB were included and classified based on plasma levels of FVIII or FIX as having severe, moderate, or mild hemophilia (< 1%, 1%–5%, and between >5 and <40% of the normal level, respectively). Hemorrhagic tendency and clinical parameters were evaluated by a hematologist.

In 37 patients of 50, the knee status was assessed by an orthopedist according to the physical joint examination score (PJEs) system (Manco‐Johnson, Nuss, Funk, & Murphy, 2000), in which the scale for the knee was from 0 (normal) to 12 (structural and functional abnormalities). Unfortunately, only in 18 patients (15 with HA and three with HB), the knee status could be assessed using MRI, since parents of the rest refused the sedation required to keep the child quiet. The 18 patients were independently examined by two radiologists using the Denver/European scoring (D/ES) (Doria et al., 2005), which included progressive (severe changes) and additive items (A‐component); both evaluated osteochondral and soft tissue conditions. The maximum scores were 16 for the osteochondral A‐component and progressive items (total score) and 4 for each of the soft tissue additive items: effusion/hemarthrosis [e], hypertrophic synovium [s], hemosiderin deposition [h] (Doria et al., 2005). Knee status of only 16 out of 18 patients was assessed using both scores (PJEs and D/ES).

Demographic, clinical, and hereditary information was obtained from all recruited individuals; also, blood samples were obtained to construct DNA bank.

### 2.2 Genotyping

The following variants were analyzed: *FV 1691G>A, FII 20210G>A*, *MTHFR 677C>T*, *MTHFR 1298A>C*, *TNFα‐308G>A*, *TNFα‐238G>A*, IL1RN*2, and *ACAN* VNTR. Genotypes were identified using PCR‐RFLP (restriction fragment length polymorphism). Because the published data for *ACAN* VNTR are absent and for IL1RN*2 VNTR limited, we genotyped 62 healthy individuals (from northern, western, and southern regions of Mexico) as a population reference, and tested the Hardy–Weinberg equilibrium (HWE).

### 2.3 Statistical analysis

Parametric data were expressed as the mean ± standard deviation and analyzed using one‐way ANOVA and *t*‐test, and nonparametric data were expressed as the median (range) and evaluated using Fisher's exact test, Mann–Whitney's *U* test, and Kruskal–Wallis test; statistical significance was considered at *p* < .05 (two‐tailed test). The concordance between radiologists was assessed using Cohen's kappa coefficient (k) and Spearman's rank correlation. In addition, allele frequencies of *ACAN* VNTR (*n* = 62) and IL1RN*2 VNTR (*n* = 58) were directly obtained using the the gene counting method in the reference group. HWE was analyzed by comparing the observed and expected genotype frequencies (Fisher's exact test). Statistical analyses were performed using SPSS v.22 for Windows (IBM).

## 3 RESULTS

Clinicopathological parameters of the patients (including those analyzed using MRI), such as disease severity (coagulant activity), patient's current age, age at first symptoms, and arthropathies number, are shown in Table 1. The patients ranged in age from 4 to 17 years (median, 13 years) and there was a tendency of age‐dependent increase in the number of affected joints: 1–3 joints were damaged in 72.5% (29/40) and 50% (4/8) of patients >8 and ≤8 years, respectively, although the differences were not significant (*p* = .38).

**Table 1:** Clinical data of the studied hemophilic patients

Clinical parameters	Hemophilia severity		
	Severe (*n* = 24)	Moderate (*n* = 15)	Mild (*n* = 10)
Coagulant activity (%), mean ± *SD*	0.5 ± 0.3	2.1 ± 0.9	6.9 ± 1.8
Median age (months) at the first symptoms (range)	8 (1–12)	7 (0–48)	36 (7–144)
Number of affected joints by clinical evaluation	0 to 3	0 to 3	0 to 1
Age at diagnosis (*n* = 49), *n* (%)			
> 12 months	5 (21)	6 (40)	7 (70)
≤ 12 months	19 (79)	9 (60)	3 (30)

Clinical parameters in patients analyzed using MRI	Severe (*n* = 10)	Moderate (*n* = 8)
Coagulant activity (%), mean ± *SD*	0.5 ± 0.4	1.8 ± 0.7
Median age (months) at the first symptoms (range)	8.5 (2–12)	7 (0–24)
Number of affected joints by clinical evaluation	1 to 3	0 to 3
Median age (months) at diagnosis (range)	12 (2–36)	11.5 (0–72)

After assessment of arthropathy using PJEs and D/ES, the patients were divided into three categories according to joint damage. Concordance analysis between the two radiologists using the progressive/additive D/ES revealed a substantial interassessment agreement only in some progressive items, and good correlation in total score, but a weak‐to‐moderate agreement in the soft tissue additive items (Table 2).

**Table 2:** Assessment of arthropathy using PJEs and D/ES

	Categories according to joint damage		
	Minimum, *n* (%)	Intermediate, *n* (%)	Severe, *n* (%)
PJEs (*n* = 37)	27 (73)	4 (11)	6 (16)
D/ES (*n* = 18)			
Radiologist 1	12 (66)	3 (17)	3 (17)
Radiologist 2	11 (61)	5 (28)	2 (11)

Concordance between radiologists using the D/ES system		
	Cohen's Kappa^a^/Spearman's coefficient^b^	*p*‐value
Progressive items: effusion, hemarthrosis, synovial hypertrophy, and subchondral cysts	0.7–1^a^	<.002
Total score (osteochondral A‐Component and progressive items)	0.640^b^	.004
Additive items e, s, and h.	0.23–0.44^a^	.00–.02

Superscript letters 'a and b' indicate when the Cohen's Kappa or Spearman coefficient were applied for statistical analysis.

Abbreviations: D/ES, Denver/European scoring; PJEs, physical joint examination score.

Genotype and allele frequencies of the biomarkers in hemophilic patients and the reference population are summarized in Table 3. Mendelian segregation analysis of the *ACAN* and IL1RN*2 VNTR polymorphisms in the reference group revealed that their genotype frequencies corresponded to HWE (*p* > .9). This is the first report on the frequency of *ACAN* VNTR polymorphisms in Mexico. No differences were observed between hemophilia patients and the reference group in *ACAN* VNTR (*p* > .10) and IL1RN*2 VNTR (*p* > .5) allele frequencies, as well as in the most common IL1RN*2 VNTR genotypes 4,4 and 4,2 (*p* = .32) or total genotypes of *ACAN* VNTR (*p* > .6). Specific IL1RN*2 allele combinations representing the minority showed different frequencies (*p* = .016) depending on the geographical origin of individuals in the reference population.

**Table 3:** Genotype and allele frequencies of biomarkers in hemophilia patients and the reference population

*ACAN* VNTR																	
Genotype frequencies																	
50 patients (*n*)	27,26	26,26	26,25	27,27	27,25	26,24	25,25	27,20	29,19	28,27	27,24	26,23	25,20	24,19	24,20	22,22	27,22
	0.26 (13)	0.16 (8)	0.01 (5)	0.1 (5)	0.06 (3)	0.06 (3)	0.06 (3)	0.04 (2)	0.02 (1)	0.02 (1)	0.02 (1)	0.02 (1)	0.02 (1)	0.02 (1)	0.02 (1)	0.02 (1)	0
62 RP (*n*)	27,26	26,26	26,25	27,27	27,24	26,24	25,25	29,27	27,25	26,17	29,26	29,25	29,24	27,22	27,21	26,21	26,20
	0.23 (14)	0.23 (14)	0.15 (9)	0.05 (3)	0.05 (3)	0.05 (3)	0.05 (3)	0.03 (2)	0.03 (2)	0.03 (2)	0.02 (1)	0.02 (1)	0.02 (1)	0.02 (1)	0.02 (1)	0.02 (1)	0.02 (1)
Allele frequencies																	
50 patients (2n)	26	27	25	24	20	22	19	29	28	23	21	17					
	0.38 (38)	0.3 (30)	0.15 (15)	0.06 (6)	0.04 (4)	0.02 (2)	0.02 (2)	0.01 (1)	0.01 (1)	0.02 (1)	0	0					
62 RP (2n)	26	27	25	24	29	21	17	22	20	28	23	19					
	0.48 (59)	0.23 (29)	0.15 (18)	0.06 (7)	0.04 (5)	0.02 (2)	0.02 (2)	0.01 (1)	0.01 (1)	0	0	0					

IL1RN*2 VNTR						
Genotype frequencies						
50 patients (*n*)	4,4	4,2	5,2	3,2	2,2	4,3
	0.48 (23)	0.33 (16)	0.08 (4)	0.06 (3)	0.06 (3)	0,02 (1)
58 RP (*n*)	4,2	4,4	2,2	4,3	5,4	3,2
	0.41 (24)	0.34 (20)	0.14 (8)	0.05 (3)	0.03 (2)	0.02 (1)
Allele frequencies						
50 patients (2n)	4	2	5	3		
	0.63 (63)	0.29 (29)	0.04 (4)	0.04 (4)		
58 RP (2n)	4	2	3	5		
	0.59 (69)	0.35 (41)	0.03 (4)	0.02 (2)		

Polymorphism (*n*)	Genotype frequencies in patients, *n* (%)		
	Homozygous (normal)	Heterozygous	Homozygous (variant)
*FV:g.1691G>A* (48)	48 (100)	0	0
*FII:g.20210G>A* (50)	49 (98)	1 (2)	0
*MTHFR:g.677C>T* (50)	12 (24)	30 (60)	8 (16)
MTHFR:g.1298A>C (50)	31 (62)	19 (38)	0
*TNFα:c.‐308G>A* (50)	38 (76)	12 (24)	0
*TNFα:c.‐238G>A* (50)	47 (94)	3 (6)	0

Note

Genotypes and alleles are arranged according to their frequencies in descending order. Bold numbers indicate genotypes and alleles.

Abbreviations: RP, reference population from northern, central, and southern Mexico. *n* = genotype count, 2*n* = allele count.

Table 4 presents analysis of association between clinical manifestations (number of affected joints and D/ES items) and informative genetic biomarkers. Parametric tests revealed that patients with the *MTHFR 677TT* genotype had a higher number of affected joints than those with the *CC* genotype, and patients with the *MTHFR 1298AC* genotype had a greater degree of effusion compared to those with the *AA* genotype (Table 4). Regarding inflammatory markers, carriers of the *TNFα‐308G>A* variant showed a higher number of subchondral cysts (according to radiologist 1) and a clear tendency to have more hemarthrosis episodes (according to radiologist 2) compared to the wild‐type group.

**Table 4:** Genotype distribution among pediatric patients with hemophilia according to clinical variables

Clinical variables	*MTHFR 677* genotypes			*p‐*value
	*CC*	*CT*	*TT*	
Number of affected joints (*n* = 47), mean ± *SD* (*n*)	0.55 ± 0.7 (*n* = 11)	1.33 ± 0.9 (*n* = 30)	1.83 ± 0.9 (*n* = 6)	.015^a^ .023^b^

	Denver/European scale	*MTHFR 1298* genotypes		
		*AA*	*AC*	
Effusion (*n* = 18), mean ± *SD* (*n*)	Radiologist 1	0.38 ± 0.52 (*n* = 8)	0.90 ± 0.31 (*n* = 10)	.043^c^
	Radiologist 2	0.50 ± 0.53 (*n* = 8)	1.20 ± 0.63 (*n* = 10)	.036^c^

		*TNFα−308* genotypes		
		*GG*	*GA*	
Number of subchondral cysts (*n* = 18), mean ± *SD* (*n*)	Radiologist 1	0.07 ± 0.26 (*n* = 14)	0.75 ± 0.95 (*n* = 4)	.041^c^
Hemarthrosis (*n* = 18), mean ± *SD* (*n*)	Radiologist 2	0.21 ± 0.42 (*n* = 14)	0.75 ± 0.50 (*n* = 4)	.051^c^

a ANOVA.

b Bonferroni (*CC* vs. *TT*).

c Mann–Whitney.

## 4 DISCUSSION

In this study, for a thrombophilic biomarker *MTHFR 677C>T*, preferential distribution of the *TT* genotype in patients with a higher number of affected joints was an unexpected finding. Several studies indicated that the *MTHFR 677C>T* polymorphism, especially in the homozygous state, could be associated with a milder clinical phenotype of hemophilia (Ahmed, Kannan, Choudhry, & Saxena, 2003; Ghosh, Shetty, & Mohanty, 2001; Nowak‐Göttl et al., 2003). However, our previous study did not reveal any attenuating effects of *MTHFR 677C>T* on the hemorrhagic phenotype in the presence of *FV 1691G>A* or *FII 20210G>A* (López‐Jiménez et al., 2009).

A study by Horvath et al. (2015) showed that the *MTHFR 677C>T* variant was correlated with low FVIII:C levels in women with HA. Consistent with this report, in our study, eight patients with the *TT* genotype had a significantly lower coagulant activity (FVIII:C or FIX:C) (1.0 ± 0.64 vs. 4.07 ± 3.4 for *CC*; *p* = .033); however, two patients had a coagulant activity of about 10%, which decreased the statistical significance of the results. The same tendency (not statistically significant) was observed in a larger group of patients (*n* = 75; data not shown). The *MTHFR 677TT* genotype alone or in combination with the heterozygous *1298AC* genotype, causes mild hyperhomocysteinemia in the presence of low folate status, while thrombotic disease seems to be primarily associated with homocystinuria (Brattström & Wilcken, 2000), which could explain the absence attenuation of hemophilia in the *MTHFR 677TT* carriers.

To date, there has been no report on the correlation between *MTHFR 1298A>C* and effusion in hemophilia or rheumatoid arthritis/inflammatory arthropathy; the observed effect of the two *MTHFR* variants should be validated in cohort studies, where patients are stratified by hemophilia severity and hemophilia causative variations in *F8* or *F9*.

The inflammatory biomarker *TNFα‐308G>A* has been associated with increased severity of rheumatoid arthritis: erosion and significant joint damage (Lee, Ji, & Song, 2007). Our results suggest a relationship between *TNFα‐308G>A* and subchondral spongy bone damage manifested by a higher number of subchondral cysts. We did not observe any effects related to the *ACAN* VNTR polymorphism, which is overall consistent with previous data on other diseases involving chronic joint degeneration, although it was suggested that the shortest allele sizes may be associated with an increased risk of cartilage degeneration in individuals where tissue degeneration has occurred much earlier than expected (Roughley et al., 2006), our results do not support this hypothesis.

PJEs and D/ES showed agreement regarding the assessment of joint damage, revealing that knee arthropathy was minimal in about 70% and 60% of patients, respectively; however, as only 16 patients were evaluated using both methods (two patients in the MRI study did not attend the physical joint examination on the date of appointment), we did not statistically analyze the concordance between PJEs and D/ES. Other systems such as the Hemophilia Joint Health score, which had good correlation with the radiological Pettersson score and moderate correlation with MRI regarding the osteochondral component, could be used. Ultrasonography is also employed to evaluate hemophilic arthropathy; however, there is insufficient evidence that it can replace MRI (Ligocki et al., 2017).

In our study, interobserver comparison of D/ES results showed substantial agreement only in some progressive items, which is different from previous results showing excellent agreement between inter‐ and intra‐observer values in both scales (Doria et al., 2005).

The knee was evaluated bilaterally, since we considered the target knee to have suffered hemarthrosis, and the other knee to have supported most of the weight, due to which damage of both joints was thought to best reflect the joint phenotype. In a previous study, joint damage (assessed by range of motion [ROM] measure) in a group of patients (*n* = 265, 75% with severe phenotype and 20% with current or history of an inhibitor) was followed‐up at 6‐month intervals for 7 years; the results indicated only the knees to show an inverse tendency, i.e. when the mean ROM score in the left knee was low, that in the right knee increased (Gomperts et al., 2017). The same authors had analyzed 13,342 genetic variants related to inflammation and immune modulation, and found 25 SNPs strongly associated with arthropathy (Gomperts et al., 2017). None of the polymorphisms related to inflammation, analyzed in this study, matched with those reported by Gomperts et al. (2017), and this could be attributed to the lower sensitivity of ROM relative to that of MRI. Besides, biomarkers related to hemorrhagic tendency and cartilage structure, considered in the present study, were not included in the study by Gomperts et al. Patients with present or past positivity for coagulation factor inhibitors were excluded in our study, in order to analyze the effect of hemorrhagic‐tendency biomarkers on joint health status. Gomperts et al. assessed this aspect by another approach, including 20% of the patients positive for FVIII inhibitors, and found a negative impact with risk of a higher ROM score (increased from 1.07 to 2.39 during the follow‐up period) in the patients with inhibitors, compared to that in negative patients.

In this study, we could not perform reliable association analysis between biomarkers and clinical parameters because of a small number of hemophilia patients and variations in coagulant activity. Although we focused on the knee as the most damaged joint, further investigation should be performed in patients who have arthropathies in other joints.

In conclusion, hemophilia patients with the *MTHFR 677TT* genotype had a higher number of affected joints than those with the other genotypes. Heterozygous patients with the *MTHFR 1298A>C* allele had a higher degree of effusion in the knee, whereas carriers of the *TNFα‐308G>A* variant showed a higher number of subchondral cysts. Cohort studies are necessary to analyze associations between genetic polymorphisms and hemophilic arthropathy detected using MRI in order to clarify genetic mechanisms underlying the clinical variability of joint damage in hemophilia.

## COMPLIANCE WITH ETHICAL STANDARDS

The study was approved by the Institutional Research and Ethics committee (number R2008‐1305‐14) in accordance with the Declaration of Helsinki. Informed consent was obtained from all participants and/or their parents or guardians.

## CONFLICT OF INTEREST

The authors declare no conflicts of interest.

## AUTHOR CONTRIBUTIONS

JJLJ and ARJC designed the study, performed the research, statistically analyzed the data, and wrote the paper; ROC performed the assessment of the knee condition by physical joint examination, and analyzed the results; HLZ and RTS evaluated the results and performed statistical analyses; ALFR, CPBM and JATP analyzed the results; JMSP performed clinical assessment of the hemophilia patients. All authors have critically reviewed and approved the final version of the manuscript.
